# Categorization of Orthologous Gene Clusters in 92 Ascomycota Genomes Reveals Functions Important for Phytopathogenicity

**DOI:** 10.3390/jof7050337

**Published:** 2021-04-27

**Authors:** Daniel Peterson, Tang Li, Ana M. Calvo, Yanbin Yin

**Affiliations:** 1 Department of Biological Sciences, Northern Illinois University, DeKalb, IL 60115, USA; danielpeterson0530@gmail.com; 2 Nebraska Food for Health Center, Department of Food Science and Technology, University of Nebraska–Lincoln, Lincoln, NE 68588, USA; tli14@unl.edu

**Keywords:** Ascomycota, phytopathogenicity, orthologous gene groups, functional annotation

## Abstract

Phytopathogenic Ascomycota are responsible for substantial economic losses each year, destroying valuable crops. The present study aims to provide new insights into phytopathogenicity in Ascomycota from a comparative genomic perspective. This has been achieved by categorizing orthologous gene groups (orthogroups) from 68 phytopathogenic and 24 non-phytopathogenic Ascomycota genomes into three classes: Core, (pathogen or non-pathogen) group-specific, and genome-specific accessory orthogroups. We found that (i) ~20% orthogroups are group-specific and accessory in the 92 Ascomycota genomes, (ii) phytopathogenicity is not phylogenetically determined, (iii) group-specific orthogroups have more enriched functional terms than accessory orthogroups and this trend is particularly evident in phytopathogenic fungi, (iv) secreted proteins with signal peptides and horizontal gene transfers (HGTs) are the two functional terms that show the highest occurrence and significance in group-specific orthogroups, (v) a number of other functional terms are also identified to have higher significance and occurrence in group-specific orthogroups. Overall, our comparative genomics analysis determined positive enrichment existing between orthogroup classes and revealed a prediction of what genomic characteristics make an Ascomycete phytopathogenic. We conclude that genes shared by multiple phytopathogenic genomes are more important for phytopathogenicity than those that are unique in each genome.

## 1. Introduction

It is estimated that as many as 5.1 million fungal species exist [1]. These organisms exhibit a diverse range of lifestyles, reproductive methods, and morphological structures [2]. Among them, the phylum Ascomycota is one of the most diverse, with over 64,000 species [1,3]. Many of these species are saprotrophic in nature, acquiring nutrients through decaying matter and non-living sources. However, several species have been shown to possess the capacity to both infect and damage living plants [4]. Infection and destruction of crops by these pathogens causes severe economic and health impact worldwide [5].

Gaining insight into the genetic mechanisms of infection and colonization could lead to the development of preventive measures to control and reduce the negative impact of these organisms. Previous studies provided annotations of predicted functions for genes of these organisms, leading to the creation of large databases of functional annotations. These functional annotations are generated through a variety of tools including but not limited to: Gene ontology (GO) annotations, which provide functional annotations for cellular components, biological functions, and molecular functions as sorted in a logical hierarchy [6], InterPro (IPR) annotations for protein families, domains, and known functional sites [7], eukaryotic orthologous group (KOG) annotations for known functions related to eukaryote domains [8], kyoto encyclopedia of genes and genomes (KEGG) annotations for enzyme categorization and function [9], and signal peptide prediction from SignalP (SigP) annotation [10]. Using the available bioinformatics tools, further annotations can be achieved, for example, for the prediction of horizontal gene transfer (HGT) using HGT-Finder [11] and transmembrane domain (TM) likelihood using TMHMM [12].

Previous comparative studies have shown that signaling pathways and specific genes can be connected directly to pathogenesis in multiple fungi [13]. Furthermore, additional studies have contributed to elucidating the variability in Ascomycota genes such as in-depth comparative analysis of plant cell wall degrading enzymes of fungi [14]. Other studies indicated that proteins with signal peptides, also referred to as effectors or secreted proteins (SPs) are essential for pathogenesis capacity in fungi [15], and their categorization resulted in several interesting findings. For example, it was revealed that plant pathogens have a larger secretome [16]. In addition, RNase, hydrolase, transferase, and oxidoreductase activities are enriched in SPs of certain pathogenic fungi [15]. These SPs are often associated with plant colonization that both enable invasion of host tissue as well as modulation of the plant immune response [17]. Other SPs were related to the production of mycotoxins, hormones, sexual pheromones, during a host-microbe interaction [16].

With the goal of gaining further insights into the pathogenic mechanism in Ascomycetes, in the present study a comparative genomics analysis was carried out using related functional annotations. Previous studies utilized comparative methods involving sequence alignments and phylogenetic analyses through various means of available bioinformatics tools including BLASTP sequence alignment [18] and orthologous gene group determination [19]. Many pan-genome analysis strategies have been completed for prokaryotic organisms [20] and are now beginning to be applied in eukaryotes, with some studies involving sequences of *Saccharomyces cerevisiae* and the plant genus *Brassica* [21]. The software for eukaryotes is starting to surface, with tools such as Pangloss which have been utilized to determine core and accessory genomes of Ascomycetes *Yarrowia lipolytica* and *Aspergillus fumigatus* [22].

For the present study, a mixture of both strategies, orthologous gene group (or orthogroups) analysis followed by more in-depth sequence analyses, were used to develop a workflow to separate the orthogroups (specifically their component proteins) of 92 Ascomycota genomes.

## 2. Materials and Methods

### 2.1. Overview of Our Analysis Idea

These 92 Ascomycota genomes were first classified into two groups: Pathogenic fungi or non-pathogenic fungi (Table S1). Determining the plant pathogenic nature of fungi is not a straightforward process, as many fungal species are pathogenic based on opportunity and environment, where they exhibit different behaviors in other circumstances [23]. To address this issue, the JGI MycoCosm Portal was used to label each organism using curated grouping of fungi, which are assembled based on known lifestyles [24].

**Figure 1:**
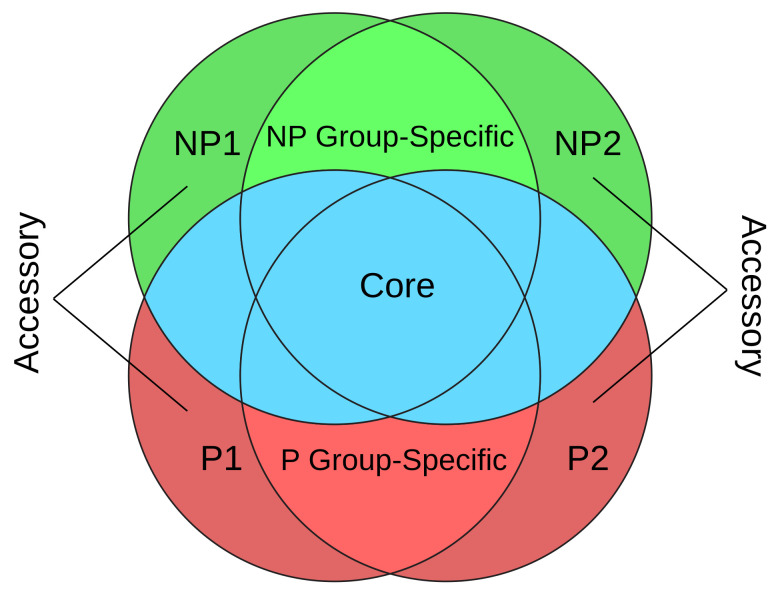
Orthologous gene group separation. Each circle represents a species and is labeled as pathogenic species (P), and non-pathogenic species (NP). P1 accessory and P2 accessory represent genome-specific pathogenic orthologous groups, NP1 accessory and NP2 accessory represent genome-specific non-pathogenic orthologous groups, P group-specific represents the group-specific orthogroups for pathogenic species, NP group-specific represents the group-specific orthogroups for non-pathogenic species. Pathogenic groups are represented in red, non-pathogenic groups in green, and core groups containing groups with both pathogenic and non-pathogenic are in blue.

Our initial separation of proteins of the 92 genomes into orthogroups was conducted using OrthoFinder [19] in a manner similar to the pan-genome analysis, which focuses on the identification of core and variable genes of a genome. However, our method differs from the pan-genome analysis in that we classify the gene content of each genome depending if the genome is pathogenic or non-pathogenic (Figure 1). By analyzing the component genes in all the orthogroups, for a pathogenic genome, its gene content was separated into three classes of orthogroups: (i) P genes are genes only found in this genome but not in the other 91 genomes (i.e., accessory genes in P genomes); (ii) P group-specific genes are genes found in at least one additional pathogenic genome but not in non-pathogenic genomes; and (iii) core genes are the rest of genes (found in at least one additional non-pathogenic genome). Similarly, the gene content of a non-pathogenic genome can be separated into NP (accessory genes in NP genomes), NP group-specific, and core classes. This idea of comparing pathogenic and non-pathogenic genomes shown in Figure 1 has been previously developed by us in bacterial genome analysis [20].

After separation of the three classes of orthogroups, enrichment analysis was then conducted on the functional annotations of the three classes of genes for each genome to look for trends in positive enrichment. Both trends and isolated occurrences of enrichment for annotation in terms of many species were found to help provide new insights for phytopathogenic Ascomycota and reinforce previous supportive information in prior studies.

### 2.2. Data Download

In total, 92 published Ascomycota genomes (Table S1) were downloaded from the JGI MycoCosm database [24] as of July 2019: 68 genomes from the curated “Plant Pathogenic Fungi” group and 24 genomes downloaded from the “Mycorrhizal Fungi” and “Endophytic Fungi” curated groups.

Functional annotation files for each genome were also downloaded from MycoCosm, if available. These included gene ontology (GO), eukaryotic orthologous group (KOG), InterPro (IPR), kyoto encyclopedia of genes and genomes (KEGG), and signal peptide (SignalP) prediction.

Full details for the downloaded data, including version numbers and strain information, can be found in Table S1.

### 2.3. Orthologous Groups and Separation

All fasta protein files were processed using OrthoFinder [19] to sort the genes into orthologous gene groups or “orthogroups”. Orthogroups were further separated using a Python script which matched the phytopathogenic nature of the species for each group’s respective proteins. Orthogroups with genes from ≥2 individual species for their respective phytopathogenicity group were designated as “group-specific”, orthogroups with ≥2 species matches in both phytopathogenicity groups were designated as “core”, and orthogroups with genes that were only from a single genome were designated as “Accessory”. Figure 1 depicts the separation of orthogroups into different classes.

**Figure 2:**
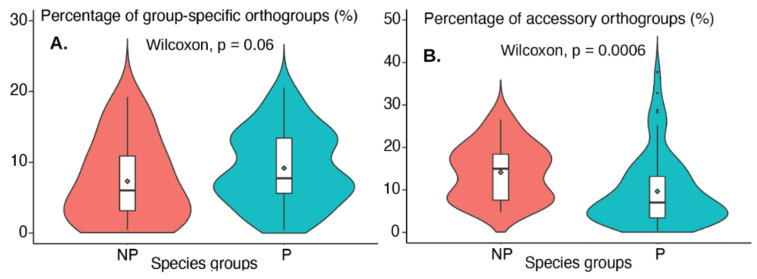
Boxplots of percentages of orthogroups in the 68 pathogenic species (P) and 24 non-pathogenic species (NP). (**A**): Group-specific orthogroups. (**B**): Accessory orthogroups. Two sample Wilcoxon tests were performed to compare the percentages between P and NP species groups. The original data for these plots can be found in Table S2.

For each genome, the percentages of core, group-specific, and accessory orthogroups were calculated and provided in Table S2. To compare these percentages between P and NP genomes, we have plotted them using boxplots in Figure 2. For example, we plotted 68 P-values and 24 NP-values side by side in Figure 2A to compare the percentages of group-specific orthogroups. The Wilcoxon test (one-tailed two sample test) was further performed on the 68 and 24 values to calculate a statistical *p*-value. A *p*-value < 0.05 means the P genomes have significantly higher percentages.

### 2.4. Transmembrane Domain (TM) Prediction

All proteins of the 92 genomes were predicted for the likelihood of being a transmembrane (TM) protein using TMHMM [12] with default parameters. Proteins with at least one predicted transmembrane segment were considered TM and used for the analysis.

### 2.5. Horizontal Gene Transfer (HGT) Prediction

All the genomes were analyzed using the DIAMOND blastp command [25] against the NCBI non-redundant (nr) database. The results output was processed using HGT-Finder [11] to determine genes most likely acquired from the horizontal gene transfer. Transfer index statistics were corrected for multiple comparisons using an included R script for false discovery rate (FDR) correction. Genes with FDR < 0.01 were used for further enrichment analysis.

### 2.6. Annotation Enrichment

A custom Python script was used to connect each orthogroup’s proteins to their respective annotations and return the number of occurrences. Annotation frequencies for species orthogroups were compared using an R script utilizing binom.test comparison and p.adjust Bonferroni correction for multiple comparisons. Group-specific orthogroups and accessory orthogroups were compared to core orthogroups intra-species. This pipeline has been developed and detailed in our previous paper [20].

### 2.7. Phylogram and Heatmap Figure Construction

In total, 132 orthogroups contain a single gene from each of the 92 genomes and thus represent single-copy conserved genes. These 132 single-copy genes of each species were concatenated, and 92 concatenated sequences were aligned using MAFFT [26]. FastTree [27] was used to build a phylogenetic tree from the aligned sequences. The Interactive Tree of Life (iTOL) online visualization tool [28] was used to generate the final phylogram and heatmap for enrichment of function terms. Phytopathogenic and non-pathogenic species JGI portal names were labeled in red and green, respectively. Branch bootstrap values were indicated numerically, mid branch. Enrichment *p*-values were heatmap plotted using −log10 values with a gradient from less significant as light blue (*p*-value of 0.05, −log10 value of −1.3) to red (*p*-value of 1 × 10^−20^, −log10 value of −20) for more significant.

### 2.8. Data and Code Availability

The authors affirm that all data necessary for confirming the conclusions of the article are available from their respective owners as referenced in the text. All the JGI genome data used in this study have been published by their original authors (cited in Table S1). All the computer codes developed for this study are available on GitHub at: https://github.com/danielpeterson0530/Orthologous-Groups-Separation (accessed on 26 October 2020).

## 3. Results

### 3.1. Orthogroup Categorization Shows That ~20% Orthogroups Are Group-Specific and Accessory

Overall, 1,154,476 proteins from the 92 Ascomycota genomes were split into 140,367 orthogroups. These include three orthogroup classes (Figure 1): (i) 99,004 proteins in accessory orthogroups (P or NP), (ii) 90,638 proteins in 21,790 group-specific orthogroups (P group-specific or NP group-specific), and (iii) 964,834 proteins in 19,573 core orthogroups (Table S2). On average, roughly 80.5% of each genome’s orthogroups was sorted into core orthogroups, 8.7% into group-specific orthogroups, and 10.8% into accessory orthogroups. Figure 2A shows the comparison of group-specific orthogroups percentages between P and NP genomes, and Figure 2B shows the comparison of accessory orthogroups percentages between P and NP genomes. It appears that the P species group tends to have a higher percentage of group-specific orthogroups (*p*-value = 0.06) but lower percentage of accessory orthogroups (*p*-value = 0.0006) than NP genomes.

Investigating the protein lengths of different orthogroup classes in each species found significant differences: Core orthogroups have the highest length, followed by group-specific orthogroups, and shortest in accessory orthogroups for a majority (91 out of 92) of the species (see Table S3 for wilcox.test *p*-values). However, the GC content did not show very significant differences: In 57 out of 92 species core orthogroups have higher GC than group-specific orthogroups, and in 39 out of 92 species group-specific orthogroups have higher GC than accessory orthogroups (see Table S3 for wilcox.test *p*-values). These results are consistent with previous studies, which showed that the less conserved orphan genes in fungi and bacteria tend to have smaller sizes and lower GC content [29,30,31].

### 3.2. Species Phylogeny Reveals That Phytopathogenicity Is Not Phylogenetically Determined

**Figure 3:**
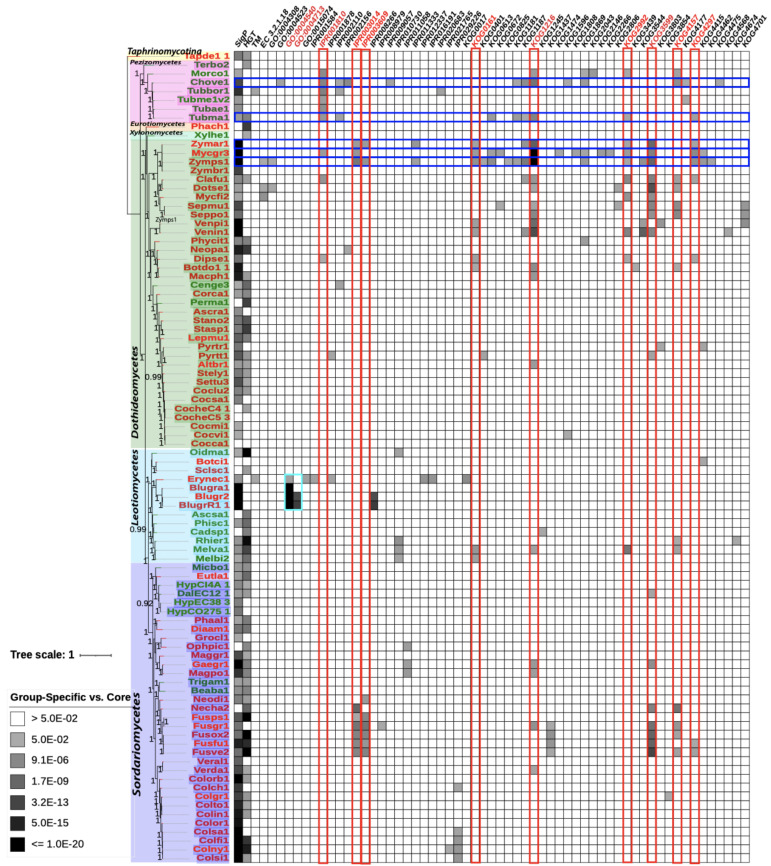
Species phylogeny and functional enrichment heatmap of group-specific orthogroups vs. core orthogroups. Bootstrap values are indicated mid branch. Annotation blocks for each species are gradient colored from white as lower significance (*p*-value of 0.05, −log10 value of −1.3) to black as higher significance (*p*-value of 1 × 10^−20^, −log10 value of 20). Pathogenic species font is indicated in red, and non-pathogenic species font is shown in green. Colored backgrounds for species portals represent their respective classes: Dark Green = Dothideomycetes, Purple = Sordariomycetes, Teal = Leotiomycetes, Pink = Pezizomycetes, Light Green = Xylonomycetes, Orange = Eurotiomycetes, and Yellow = Taphrinomycetes. Selected functional terms and species are highlighted with colored frames as outlined in the main text: Red—top enriched group-specific functional terms in Table 1, blue—five most enriched species in Table 2, and cyan—four species with two enriched GO terms (discussed in the text). Abbreviated annotations are as follows: SigP: Signal peptide; TM: Transmembrane domain; HGT: Horizontal gene transfer; KOG: Eukaryotic orthologous group database; IPR: Integrated protein resource database; EC: Enzyme database.

**Figure 4:**
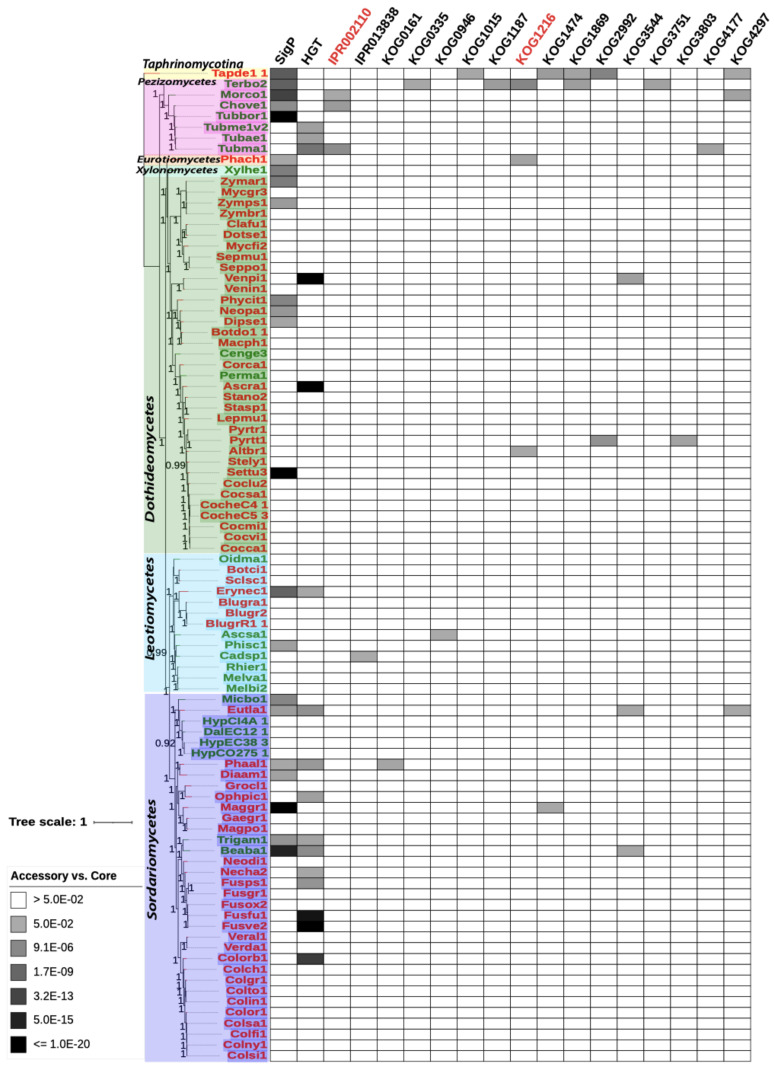
Species phylogeny and functional enrichment heatmap of accessory orthogroups vs. core orthogroups. See the Figure 3 legend for details.

Figure 3 and Figure 4 depict the phylogeny (same in the two figures) for the concatenated single-copy genes of all Ascomycota species included in our analysis (see Methods in Section 2). Of the 92 Ascomycota species, 91 are of the subphylum Pezizomycotina, and one of the subphylum Taphrinomycotina (Tapde1_1, used as the outgroup for other species). According to the NCBI taxonomy, the 91 Pezizomycotina subphylum species can be further divided into classes: 35 Dothideomycetes, 34 Sordariomycetes, 13 Leotiomycetes, seven Pezizomycetes, one Xylonomycetes, and one Eurotiomycete, which are shown with different background colors in the phylogeny.

Of all the species, the most distant in the analysis based on branch length (Figure 3 and Figure 4) were *Taphrina deformans* (JGI portal ID: Tapde1_1), *Terfezia boudieri* (Terbo2), *Morchella importuna* (Morco1), *Phaeomoniella chlamydospora* (Phach1), and *Xylona heveae* (Xlyhe1). These species are from the classes Pezizomycetes (Terbo2 and Morco1), Xylonomycetes (Xlyhe1), Eurotiomycetes (Phach1), and Taphrinomycotina (Tapde1_1) showing the largest phylogenetic distance from other species in the analysis. This is consistent with current consensus phylogenies of fungi [32] which depicts these classes as generally smaller and more distant than Dothideomycetes and Sordariomycetes.

Notable on the phylogram, phytopathogenic (red font in Figure 3 and Figure 4) and non-phytopathogenic (green font) species do not appear separately clustered. For example, the Dothideomycetes class contains two non-phytopathogenic species, while others are phytopathogenic. A recent paper focusing on the comparative analysis of *Dothideomycetes* genomes has made a similar observation [33]. This suggests that phytopathogenic and non-phytopathogenic species can be inter-converted between phylogenetically related species through the acquisition or loss of pathogenicity genes by various means. For example, the cereal pathogen *Fusarium pseudograminearum* genome contains genes horizontally transferred from other fungal and bacterial pathogens of cereals, knocking out these genes has been shown to significantly reduce virulence of *F. pseudograminearum* [34]. 

### 3.3. Group-Specific Orthogroups Have More Enriched Functional Terms than Accessory Orthogroups

Figure 3 and Figure 4 also showed the statistically enriched functional terms of GO, KOG, IPR, KEGG, SignalP, TMHMM, and HGT-Finder in group-specific orthogroups and accessory orthogroups, respectively with the core orthogroups as the background data for statistical tests (functional enrichment tests using the binom.test in R, see Section 2.6). Specifically, for each functional term in each species, the number of occurrences of core orthogroups, of accessory orthogroups, and of group-specific groups were calculated (Tables S4 and S5). Functional enrichment tests were then performed, and such tests could reveal, comparing against the core orthogroups, which functional terms are statistically significantly over-represented in accessory orthogroups and group-specific groups. In Figure 3 and Figure 4, group-specific orthogroups showed that 61 individual annotation terms were significantly enriched (adjusted *p*-value < 0.05) for at least one species when compared against the number of occurrences in a species core orthogroups. In accessory orthogroups, 18 individual annotation terms were significantly enriched. In addition, 13 terms were significantly enriched in both group-specific and accessory orthogroups.

Phylogenetically distant species in the analysis, *Taphrina deformans* (Tapde1_1) and *Terfezia boudieri* (Terbo2), showed a significant enrichment of six annotation terms in accessory orthogroups in Figure 4. This is expected as these Ascomycota species were phylogenetically more distant to other species in the analysis, and therefore more unique genes tend to be found in their genomes.

### 3.4. Secreted Proteins with Signal Peptides Show Higher Significance in Group-Specific Orthogroups than in Accessory Orthogroups

Group-specific orthogroups shown in Figure 3 present more species and higher significance of enrichment for signal peptides when compared to accessory orthogroups in Figure 4. Occurrence was greater in group-specific orthogroups, with 60/68 total (88.2%) phytopathogenic species and 19/24 total (79.2%) non-phytopathogenic species having significantly enriched signal peptides. Accessory orthogroups showed 13/68 total (19.1%) phytopathogenic species and 9/24 total (37.5%) non-phytopathogenic species having significantly enriched signal peptides.

All the species group-specific orthogroups had an overall average adjusted *p*-value of 0.04 for signal peptide enrichment, with average adjusted *p*-values of 0.022 for exclusively phytopathogenic species and 0.091 for exclusively non-phytopathogenic species. This suggests that enrichment for signal peptides is more significant for phytopathogenic species than non-phytopathogenic species. For all species accessory orthogroups had an overall average adjusted *p*-value of 0.62, with average adjusted *p*-values of 0.68 for exclusively phytopathogenic species and 0.46 for exclusively non-phytopathogenic species. Therefore, the secreted proteins with signal peptides are more likely to be found in the more conserved genes shared by multiple phytopathogenic species. Numbers for enrichment of signal peptides for group-specific orthogroups can be found in Table S4.

### 3.5. HGTs Show Higher Significance and Higher Occurrence in Group-Specific Orthogroups than in Accessory Orthogroups

Similar to secreted proteins, horizontally transferred genes also have more occurrence and higher significance in group-specific orthologous groups (Figure 3) than in accessory orthogroups (Figure 4). Group-specific orthogroups had significantly enriched HGT annotations in 38/68 (55.9%) phytopathogenic species and 17/24 (70.8%) non-phytopathogenic species. Accessory orthogroups had 11/68 (16.2%) phytopathogenic species and 5/24 (20.8%) non-phytopathogenic species having significantly enriched HGT annotations.

All the species group-specific orthogroups had an overall average adjusted *p*-value of 0.15 for HGT enrichment, with average adjusted *p*-values of 0.16 for phytopathogenic species and 0.11 for non-phytopathogenic species. Therefore, there is not much difference in terms of the importance of HGT in phytopathogenic species and non-phytopathogenic species. All species accessory orthogroups had an overall average adjusted *p*-value of 0.60, with average adjusted *p*-values of 0.58 for phytopathogenic species and 0.63 for non-phytopathogenic species. Numbers for enrichment of signal peptides for accessory orthogroups can be found in Table S5.

### 3.6. Phytopathogenic Fungi Have More Enriched Functional Terms in Group-Specific Orthogroups than Accessory Orthogroups

Comparing Figure 3 and Figure 4, all genomes showed enrichment of a higher number of functional terms for group-specific orthogroups (90 out 92 genomes with 62 significantly enriched terms) over accessory orthogroups (37 out 92 genomes with 18 significantly enriched terms). This was anticipated, as group-specific orthogroups are more conserved (contain proteins from multiple genomes) than accessory orthogroups (contain one or multiple proteins from one genome). This trend is particularly evident in phytopathogenic fungi, where 66 genomes have 52/62 (83.9%) enriched functional terms in group-specific orthogroups. As a comparison, 24 non-pathogenic genomes have 26/62 (41.9%) enriched functional terms in group-specific orthogroups. For accessory orthogroups, in phytopathogenic fungi, 23 genomes have 11/18 (61.1%) enriched functional terms, and 14 non-pathogenic genomes have 13/18 (72.2%) enriched functional terms.

Furthermore, Table 1 provides a list of functional terms that are significantly enriched in group-specific orthogroups ranked by the number of species (also highlighted in Figure 3). This list shows that secreted proteins with signal peptides (Signal Pep) and horizontally transferred genes (HGT) are two functional terms that appear in the majority of studied genomes in terms of the enrichment in group-specific orthogroups.

### 3.7. More Phytopathogenic Fungi than Non-Phytopathogenic Fungi Are Found to Have a Larger Number of Enriched Functional Terms in Group-Specific Orthogroups

Unlike accessory orthogroups, group-specific orthogroups are less affected by the genome sampling, since group-specific orthogroups contain at least two genomes. Ranking the 92 genomes based on the number of enriched functional terms found that eight of the top 10 genomes are phytopathogenic fungi (Table 2). For example, the grass pathogen *Zymoseptoria pseudotritici* (Zymps1) [35] contains 21 enriched terms, and its closely related wheat pathogen *Mycosphaerella graminicola* (Mycgr3) [36] contains 16 enriched terms. Not surprisingly, these top ranked genomes tend to have a higher percentage of group-specific orthogroups (Zymps1: 18.71% and Mycgr3: 14.75%, Table S2), so that more genes were used for enrichment analysis resulting in more significantly enriched functional terms.

## 4. Discussion

In this study, we have analyzed 92 published Ascomycota genomes to identify genes and functions putatively involved in the phytopathogenicity in fungi. This has been mainly achieved by employing categorizing orthologous gene groups and separating phytopathogenic and non-phytopathogenic genomes. The most significant finding is that genes shared by multiple phytopathogenic genomes are more important for phytopathogenicity than those that are unique in each genome, which agrees with our previous finding made in pathogenic bacteria [20]. 

Another very interesting finding is that secreted proteins with signal peptides are significantly enriched in group-specific orthogroups in most species. Out of the 92 genomes, 79 (85.9%) have signal peptide significantly enriched in their group-specific orthogroups (Table 1), compared to only 22 (23.9%) genomes having signal peptide significantly enriched in their accessory orthogoups. The higher significance of enrichment of secreted proteins with signal peptides in group-specific orthogroups (Figure 3 and Figure 4) suggests that they are more conserved among Ascomycota fungi, rather than being unique to individual species. Secreted proteins play an important role in host tissue invasion and modulation of plant defenses, often referred to as effectors for their roles during plant colonization [17]. Certain effectors have been described as the product of more recent acquisition through means of horizontal gene transfer between fungi, such as major virulence gene *ToxA* in *Pyrenophora tritici-repentis* [37]. Other effector genes, such as several avirulence genes (AVR) of *Magnaporthe oryzae* and other species, have shown a long history of co-evolution with plant resistance (R) genes [38]. Many signatures of fast evolution have been observed in effector genes of most pathogens through comparative analyses [39], with effectors often located in flexible genomic regions, making them more susceptible for host jumps and events related to gain, loss, and diversification in fungi [40]. Previous studies have shown that signal peptides effectors in Ascomycota are functionally related to host colonization [17]. Our results reveal a higher percentage of occurrence for significant enrichment of signal peptides in the group-specific orthogroups of phytopathogenic and non-phytopathogenic species (Table 1). As our non-phytopathogenic group is constructed of both endophytic and mycorrhizal species, which maintain the capacity for host colonization, we would expect to see enrichment for signal peptides in these species, as well.

In addition, HGTs are also more prevalently enriched in group-specific orthogroups than accessory orthologous groups. Notably, HGT is a driving factor for lineages to acquire novel traits for competitive advantage in the environment [41,42]. Our results indicated that horizontal gene transfer (HGT) annotations showed higher significance and higher occurrence in group-specific orthogroups than accessory orthogroups (Figure 3 and Figure 4), and that non-phytopathogenic species had a higher occurrence and average significance than phytopathogenic species (Table 1). HGT in eukaryotes plays a major role in the development of fungal pathogenesis [42] and it has been more recognized as a driving factor in the evolution of plant pathogenesis for fungal lineages [43]. It should be mentioned that HGTs of this study were identified by the HGT-Finder [11], implementing a heuristic algorithm to analyze the atypical phyletic distribution of BLAST hits. Although this algorithm is favored for a genome-scale HGT screening, more rigorous phylogenetic analyses of individual genes will be needed to further validate these HGTs and reveal deeper insights regarding the timeframe of possible HGT genes in these fungi. In addition, the role and impact of HGT in endophytic and mycorrhizal fungi is not well understood, and it is only recently becoming a subject of study [41].

Aside from secreted proteins with signal peptide and HGTs, there are also other functional terms (Table 1) that demonstrated a significant enrichment in group-specific orthogroups in a number of species. Some of these terms were enriched in a higher percentage of phytopathogenic fungi. For example, KOG1216, von Willebrand factor, and related coagulation proteins, are significantly enriched in 16 pathogenic species and four non-pathogenic species, while accessory orthogroups showed two pathogenic species and one endophytic non-pathogenic species. The von Willebrand A (VWA) domain is well known to be found in cellular adhesion and extracellular matrix (ECM) proteins and is highly conserved in Eukaryotes, Eubacteria, and Archaea [44]. The fungus *Mycosphaerella graminicola* (*Septoria tritici*) is known to have high levels of expression for von Willebrand factor annotated transcripts related to cellular adhesion during infection, where it causes leaf blotch of wheat [45].

KOG3599 is a family of Ca^2+^-modulated nonselective cation channel polycystin. It had an average adjusted *p*-value of 1.32 × 10^−3^ between sixteen pathogenic species and one non-pathogenic species (Table 1), while accessory orthogroups showed no significantly enriched orthogroups in any species. In a previous study, *Zymoseptoria tritici* showed higher expression of Ca^2+^-modulated nonselective polycystin proteins and proteins related to signal transduction cascades and activation of defense/anti-oxidative stress responses in an incompatible interaction of wheat [46]. Certain plants utilize Ca^2+^ pathways to help mediate stress responses [47] where an invading fungus in a biotrophic stage would want to prevent the degradation of cell wall polysaccharides and stabilize the plant cell wall [46]. Our observation of higher enrichment for this functional term is congruent with these reports, given the importance of cellular adhesion and mediation of stress response during plant colonization by fungi.

The InterProt annotation for IPR001810 for cyclin-like F-box domain showed significant enrichment in group-specific orthogroups for three pathogenic species and six non-pathogenic species of the *Tuber* genus (Table 1). This conserved domain functions in protein binding and to aid in ubiquitination to mark proteins for degradation [48]. A recent study identified this domain in selected open reading frames (ORFs) of the *Alternaria alternata Alt* locus, which were involved in the infection of apples and showed high similarity to genes involved in plant defense responses [49]. Certain bacterial pathogens are also known to utilize eukaryotic F-box effectors to promote proliferation in their hosts [50]. Given the highly conserved nature of this domain and lack of supportive material related to possible roles in eukaryotic pathogenicity, it is unclear whether there is a direct link.

Two gene ontology terms: GO:0004540 (ribonuclease activity) and GO:0004713 (protein-tyrosine kinase activity) showed high levels of enrichment in three phytopathogenic *Blumeria graminis* (BlugrR1_1, Blugr2, Blugra1) group-specific orthogroups (cyan frame in Figure 3). A previous study of *B. graminis* suggests that an ancestral secreted ribonuclease spurred a complement of secreted proteins, and that these proteins are involved in the evolution of grass and cereal powdery mildew, as well as numerous proteins that act as important effectors of pathogenicity for the purposes of suppressing host defense [51]. Fungi, such as the genus *Aspergillus*, can utilize highly specific extracellular ribonucleases for what is suspected to be cytotoxic activities related to defense and parasitism [52]. Given these insights, there is a possible correlation between extracellular ribonucleases of *B. graminis* and its pathogenicity. Moreover, given that the protein tyrosine kinase (PTK) activity is known to be essential to cell proliferation, cell differentiation, immune response, organ development, and many other cellular processes [53], PTK could also be important in the capability of phytopathogens to invade the plant host.

Lastly, a higher percentage of accessory orthogroups was found in more distant species. In Figure 4, species more phylogenetically distant from the others tended to have higher numbers of enriched accessory orthogroups (e.g., Tapde1_1 and Terbo2). This observation was expected, as genes predicted to share more sequence identity with others should be more likely to be categorized into a shared orthogroup. This trend of higher accessory orthogroup categorization in distant organisms is most likely due to the current genome availability, where our sample of 92 species exhibited varying ranges of relationships to one another. It would be expected that with additional genomes included in the analysis, that percentages of accessory orthogroups would decrease and core and group-specific orthogroups categories would increase for more distantly related species. This limitation can and will be improved in time with the addition of more closely related species to distant organisms, such as those from *Taphrinomycetes*.

## 5. Conclusions

By a comparative genomics analysis of 92 published Ascomycota genomes, we conclude that genes shared by multiple phytopathogenic genomes are more important for phytopathogenicity than those that are unique in each genome. We also showed evidence that phytopathogenicity is not phylogenetically determined. Our results revealed a list of positively enriched functional terms that are significantly over-represented in pathogen-specific orthogroups. These functional terms include not only secreted effector proteins and horizontally transferred proteins that are previously known to be important for fungal phytopathogenicity, but also other unknown functions that may make an Ascomycete phytopathogenic.

## Figures and Tables

**Table 1:** Top functional terms significantly enriched in group-specific orthogroups in at least eight species.

Enriched Terms	Description	# of Species (% of Total 92)	# of P Species (% of Total 68)	# of NP Species (% of Total 24)
*Signal Pep*	Signal peptide	79 (85.9%)	60 (88.2%) *	19 (79.2%)
*HGT*	horizontal gene transfer	55 (59.8%)	38 (55.9%)	17 (70.8%)
*KOG1216*	Von Willebrand factor and related coagulation proteins	20 (21.7%)	16 (23.5%) *	4 (16.7%)
*KOG3599*	Ca^2+^-modulated nonselective cation channel polycystin	17 (18.5%)	16 (23.5%) *	1 (4.2%)
*KOG4157*	beta-1,6-N-acetylglucosaminyltransferase, contains WSC domain	13 (14.1%)	9 (13.2%)	4 (16.7%)
*KOG2992*	Nucleolar GTPase/ATPase p130	10 (10.9%)	7 (10.3%)	3 (12.5%)
*IPR001810*	Cyclin-like F-box	9 (9.8%)	3 (4.4%)	6 (25.0%)
*IPR003014*	N/apple PAN	9 (9.8%)	9 (13.2%) *	0
*KOG0161*	Myosin class II heavy chain	9 (9.8%)	7 (10.3%) *	2 (8.3%)
*IPR003609*	Apple-like	8 (8.7%)	8 (11.8%) *	0
*KOG4297*	C-type lectin	8 (8.7%)	7 (10.3%) *	1 (4.2%)

* These percentages are higher in P (phytopathogenic) species than in NP species. All these terms are highlighted in Figure 3.

**Table 2:** Top 10 species with the most functional terms significantly enriched in group-specific orthogroups.

JGI ID *	Species	Species Type	Taxonomy Class	# of Enriched Terms
*Zymps1*	*Zymoseptoria pseudotritici*	P	*Dothideomycetes*	21
*Mycgr3*	*Mycosphaerella graminicola*	P	*Dothideomycetes*	16
*Chove1*	*Choiromyces venosus*	NP	*Pezizomycetes*	14
*Tubma1*	*Tuber magnatum*	NP	*Pezizomycetes*	10
*Zymar1*	*Zymoseptoria ardabiliae*	P	*Dothideomycetes*	10
*Clafu1*	*Cladosporium fulvum*	P	*Dothideomycetes*	9
*Erynec1*	*Erysiphe necator*	P	*Leotiomycetes*	9
*Fusgr1*	*Fusarium graminearum*	P	*Sordariomycetes*	8
*Fusve2*	*Fusarium verticillioides*	P	*Sordariomycetes*	8
*Venin1*	*Venturia inaequalis*	P	*Dothideomycetes*	8

* The top five species are highlighted in Figure 3.

## Data Availability

The authors affirm that all the data necessary for confirming the conclusions of the article are available from their respective owners as referenced in the text. All the JGI genome data used in this study have been published already. All the computer codes developed for this study are available on GitHub at: https://github.com/danielpeterson0530/Orthologous-Groups-Separation (accessed on 26 October 2020).

## Supplementary Materials

The following are available online at https://www.mdpi.com/article/10.3390/jof7050337/s1, Table S1: Ascomycota scientific names, JGI group, JGI portal identifiers, and Pubmed ID(s) for fungi used in this study; Table S2: Distribution of orthologous groups in each Ascomycota species; Table S3: Wilcox test *p*-values to compare the GC content and protein sequence length between core and group-specific, and between group-specific and accessory orthogroups; Table S4: Statistically enriched functional terms in group-specific orthogroups vs. core orthogroups; Table S5: Statistically enriched functional terms in accessory orthogroups vs. core orthogroups.
